# Intra-Patient Heterogeneity of Circulating Tumor Cells and Circulating Tumor DNA in Blood of Melanoma Patients

**DOI:** 10.3390/cancers11111685

**Published:** 2019-10-29

**Authors:** Katharina Gorges, Lisa Wiltfang, Tobias M. Gorges, Alexander Sartori, Lina Hildebrandt, Laura Keller, Beate Volkmer, Sven Peine, Anna Babayan, Ingrid Moll, Stefan W. Schneider, Sören Twarock, Peter Mohr, Jens W. Fischer, Klaus Pantel

**Affiliations:** 1 Institute for Pharmacology and Clinical Pharmacology, University Hospital of the Heinrich-Heine-University, 40225 Düsseldorf, Germany; roeck@hhu.de (K.G.); soeren.twarock@hhu.de (S.T.); jens.fischer@uni-duesseldorf.de (J.W.F.); 2 Department of Tumor Biology, University Medical Center Hamburg-Eppendorf, 20251 Hamburg, Germany; l.wiltfang@uke.de (L.W.); t.gorges@uke.de (T.M.G.); l.keller@uke.de (L.K.); a.babayan@uke.de (A.B.); 3 Agena Bioscience GmbH, 22761 Hamburg, Germany; alexander.sartori@agenabio.com; 4 Department of Dermatology, University Medical Center Hamburg-Eppendorf, 20251 Hamburg, Germany; l.hildebrandt@uke.de (L.H.); st.schneider@uke.de (S.W.S.); 5 Department of Dermatology, Elbe Kliniken, 21614 Buxtehude, Germany; beate.volkmer@elbekliniken.de (B.V.); peter.mohr@elbekliniken.de (P.M.); 6 Institute of Transfusion Medicine, University Medical Center Hamburg-Eppendorf, 20246 Hamburg, Germany; s.peine@uke.de; 7 DermatoMed, 22303 Hamburg, Germany; dermatomed@arztzentrum.de

**Keywords:** melanoma, ctDNA, CTC, liquid biopsy

## Abstract

Despite remarkable progress in melanoma therapy, the exceptional heterogeneity of the disease has prevented the development of reliable companion biomarkers for the prediction or monitoring of therapy responses. Here, we show that difficulties in detecting blood-based markers, like circulating tumor cells (CTC), might arise from the translation of the mutational heterogeneity of melanoma cells towards their surface marker expression. We provide a unique method, which enables the molecular characterization of clinically relevant CTC subsets, as well as circulating tumor DNA (ctDNA), from a single blood sample. The study demonstrates the benefit of a combined analysis of ctDNA and CTC counts in melanoma patients, revealing that CTC subsets and ctDNA provide synergistic real-time information on the mutational status, RNA and protein expression of melanoma cells in individual patients, in relation to clinical outcome.

## 1. Introduction

Malignant Melanoma is the deadliest of all skin cancers and accounted for more than 59,000 deaths worldwide in 2015 [1]. In recent years, systemic treatment of metastatic melanoma has been transformed. Improved understanding of the genetic landscape of melanoma led to the development of BRAF and MEK inhibitors for patients with BRAF mutated tumors [2,3]. However, the frequently profound response to BRAF/MEK inhibition is transient in about 50% of all cases. Additional therapeutic options were derived from insights into the molecular controls of the immune system. CTLA-4 and PD-1/PDL-1 neutralizing antibodies are used independent of mutational status, leading to a durable response. However, this only occurs in a subset of patients [4,5]. 

Hence, reliable biomarkers, which allow the prediction of therapeutic response and/or the development of therapeutic resistance as early as possible, are urgently needed. To date, tissue biopsies have predominantly been utilized to achieve this goal. However, repeated biopsies to study the frequently adapting heterogeneous tumor cell populations in melanoma are invasive, difficult to obtain and may not represent the entire molecular tumor profile [6,7,8]. 

Circulating tumor cells (CTCs), as well as circulating tumor DNA (ctDNA), are shed into the bloodstream from either primary or metastatic lesions. Serial analysis of liquid biopsies might provide a dynamic and minimally invasive option to screen the pathological characteristics of the entire tumor, based on a simple blood withdrawal [9,10]. PCR-based studies on melanoma-associated antigens (MAAs, e.g., *MART-1*, *MAGE-A3*, *PAX3*, and *GM2/GD2*)—that are not present in leukocytes—have shown that their presence was correlated with an advanced patient stage, as well as decreased disease-free and overall survival rates, in several studies [11,12,13,14]. In addition, an increased quantity of ctDNA was found to be prognostic in melanoma patients and might provide useful information on the mutational status of the disease [15,16]. Furthermore, ctDNA might serve as a surrogate marker of tumor burden in metastatic melanoma patients [17]. Although promising, the information presented by these assays is still limited in its capability to advise therapeutic decisions, since it is based on the analysis of a pooled cell fraction (healthy and tumor tissue). 

However, the enrichment of melanoma CTCs has been challenging, mainly due to their large molecular heterogeneity (e.g., surface marker expression and cellular size). To date, the use of either marker or size-based enrichment methods leads to the loss of surface marker negative or small CTCs, respectively. Interestingly, evidence is accumulating that the mutational heterogeneity of melanoma cells might influence the cellular expression of surface markers as well as their cellular volume. For example, activation of the RAS/RAF pathway drives the expression of CSPG4, the most commonly used surface protein, to enrich melanoma CTCs [18,19]. BRAF-inhibition decreases the cellular volume of enlarged BRAF-mutated melanoma cells in a glucose dependent manner [20]. The identification of all CTC subpopulations might, therefore, be pivotal for the correct stratification of patients and subsequent therapeutic decisions. In addition to diagnostic applications, the detailed analysis of CTC subpopulations may yield new insights into the process of melanoma metastasis. 

Here, we show that the cell surface marker expression of melanoma cells depends on their mutational status. We provide a novel enrichment approach, which allows the isolation and complete molecular analysis of different CTC subpopulations and ctDNA analysis from one blood sample. In addition, we demonstrate how combined CTC and ctDNA analyses can reveal synergistic information, which is potentially relevant for personalized therapy in metastatic melanoma. 

## 2. Results

### 2.1. RAS/RAF Activating Mutations Lead to A Distinct Melanoma Marker Expression Pattern

Activation of the RAS/RAF pathway has been suggested to increase the expression of the melanoma surface marker CSPG4 in neural cells [18]. Here, we tested whether this is also the case for the expression of the previously described [21,22] melanoma-specific genes, by analyzing The Cancer Genome Atlas (TCGA) (https://www.cancergenome.nih.gov/) database. Melanomas containing RAS/RAF activating mutations were compared to melanomas without RAS/RAF activating mutations. 

**Figure 1:**
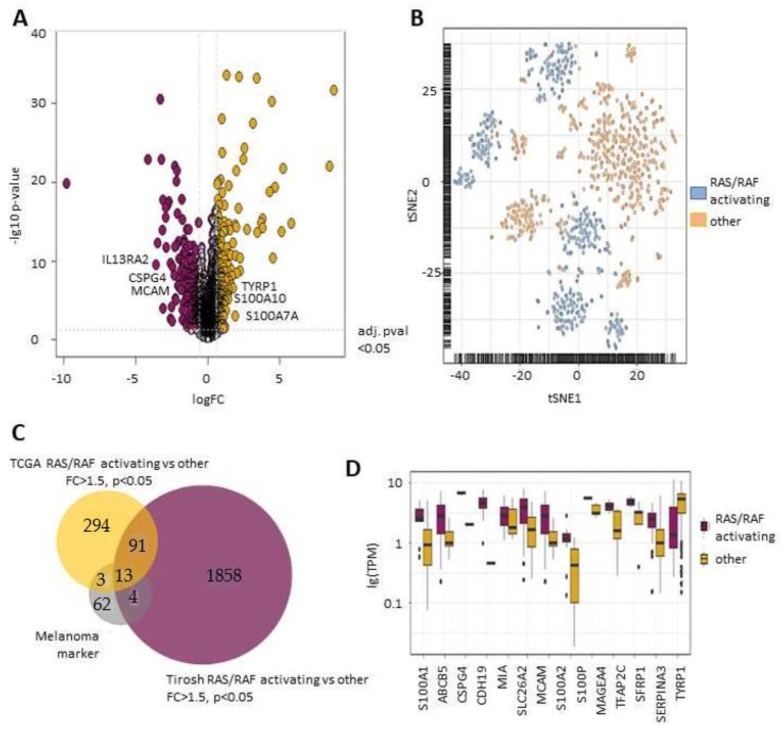
BRAF/NRAS mutations determine cellular marker expression in malignant melanoma. (**A**) Volcano plot of differentially regulated genes in the TCGA data set, comparing melanomas containing BRAF/NRAS mutations (RAS/RAF activating) and not BRAF/NRAS mutated melanomas (other). Significantly reduced genes (<lgFC − 1) are depicted in violet; significantly increased genes (>lgFC 1) in yellow (**B**) *t*-SNE plot, based on melanoma markers expressed on single cells (Tirosh et al.), derived from BRAF/NRAS mutated tumors and not BRAF/NRAS mutated tumors. (**C**) Venn diagram of all differentially expressed genes between RAS/RAF activating and other tumors of the TCGA and Tirosh cohorts and specific melanoma marker genes. (**D**) Significantly differentially expressed melanoma marker genes in the Tirosh data set. * = *p* < 0.05.

In total, 16 melanoma marker genes (S100A1, ABCB5, CDH19, MIA, SLC26A2, MCAM, S100A2, S100P, MAGEA4, TFAP2C, SFRP1, SERPINA3, CSPG4, TYRP1, IL13RA2, S100A7A) were highly expressed in melanomas with activating mutations (Figure 1A,C). We next analyzed whether those differentially expressed genes could be detected on single cell level within each group. For this purpose, data from 2056 single melanoma cells, derived from the metastatic tumors of 19 melanoma patients, were analyzed [21]. *t*-SNE clustering clearly demonstrated that cells harboring a RAS/RAF activating mutation can be differentiated from cells without activating mutations, based on melanoma marker genes (Figure 1B). In total, 13 melanoma marker genes were specifically increased in the RAS/RAF cohort in both the bulk tumor (TCGA) and single melanoma cell cohort [21] (Figure 1C). These markers include the two most commonly used melanoma surface markers for CTC enrichment, CSPG4 and MCAM (Figure 1D).

### 2.2. A Combined Enrichment Approach Allows the Detection of CTC Subpopulations

**Figure 2:**
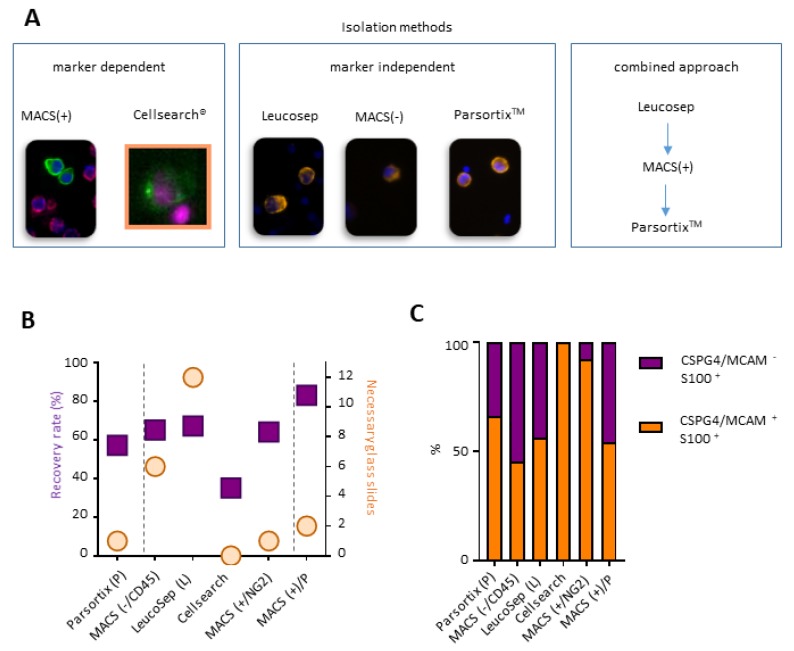
Method to analyze melanoma cell subsets. (**A**) Tested isolation methods for the membrane marker dependent and independent enrichment of melanoma cells. (**B**) Recovery rate of 50 melanoma cells in 7.5 mL of blood and subsequent histological slides needed for analysis. (**C**) Amount of melanoma cells positive for membrane markers CSPG4/MCAM and intracellular S100. *N* = 3–4.

We reasoned that differential marker expression between mutational melanoma subsets might distort clinical decisions based on CTC counts and characteristics, depending on the utilized enrichment method. To identify a method which would (i) allow the detection of both marker positive and marker negative cells and (ii) enable a thorough molecular analysis of the isolated single cells (e.g., DNA, RNA, immunocytochemistry), we tested two marker dependent, three marker independent and one combined approach, for CTC detection (Figure 2A). The recovery rate for each method was determined by spiking 25 individually micro-manipulated cells of an RAF mutated (SKMEL28) and non-RAS/RAF mutated (MeWo) cell each into 7.5 mL blood from healthy donors. The recovery rate varied between 36% (Cellsearch^®^) and 82% (combined approach) (Figure 2B). 

An additional obstacle for the translation of each method into clinical practice is provided by the number of contaminating leukocytes, represented by the number of histological slides needed to analyze cellular output after each enrichment method (1 million resulting cells were mounted onto each slide for the subsequent stain and detection of the cells). For complete analysis of a Leucosep^®^ enriched sample, an average of 12 slides was needed, whereas only one slide was necessary after CellSearch^®^ enrichment (Figure 2B). As expected, subsequent staining of the cells for CSPG4/MCAM and S100 (as a marker for surface marker negative cells) revealed that marker independent methods tend to isolate a more representative cellular population than marker dependent methods (Figure 2C). 

For the following analysis, we decided to use the combined approach, which allowed a good resolution of the melanoma cell subpopulations, and yielded a high recovery rate, a low amount of contaminating leukocytes, and a complete molecular analysis of CTCs.

### 2.3. Targeted Sequencing Reveals Mutational CTC Subclones

**Figure 3:**
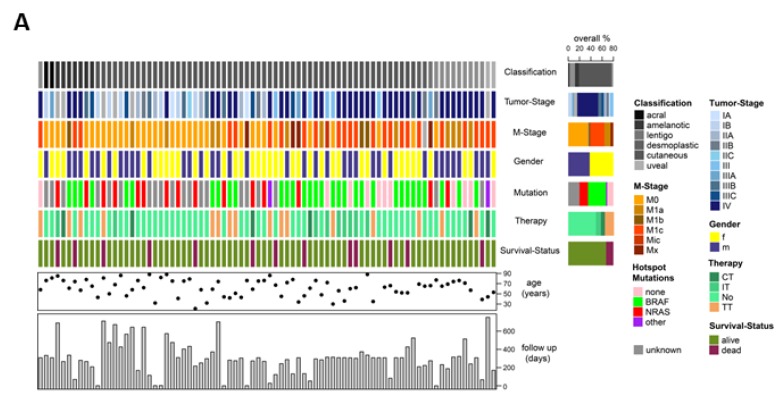

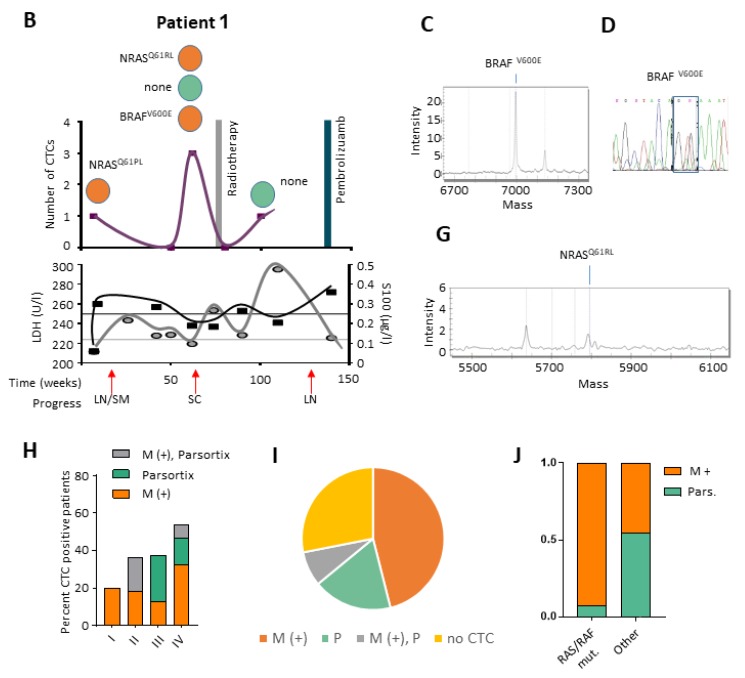
Combined enrichment method for the analysis of cellular subpopulations of circulating melanoma cells in patients. (**A**) Overview of patients with cutaneous, acral, mucosal and uveal melanoma of different disease stages, included in the study. (**B**) Time course of CTCs, enriched by positive selection (orange circles) and Parsortix™ (green circles) and their detected mutations, as well as LDH and S100 serum levels in patient #1. (**C**) Mass spectrum plot of BRAFV600E mutation and (**D**) Sanger sequencing of the same sample. (**G**) Mass spectrum plot of NRASQ61RL mutation. (**H**) Percentage of patients positive for circulating melanoma cells enriched by positive selection (MACS, M+), Parsortix™ or both. (**I**) Distribution of enriched CTCs in stage 4 patients prior to treatment. (**J**) CTCs enriched by positive selection, and Parsortix™ in NRAS/BRAF mutated patients, and not BRAF/NRAS mutated patients.

We collected whole blood samples from 84 melanoma patients receiving current standards of clinical care, to determine if CTC subtypes can be used to support clinical diagnostics. Patients presenting with stage I–IV cutaneous, acral, amelanotic, lentigo, desmoplastic or uveal melanoma were included. Patients were between 21–88 years old and received treatment, including chemotherapy, targeted therapies and immunotherapy. Some patients have been followed up for more than three years (Figure 3A). 

Overall, 32% (27 patients, Supplementary Table S1) of all patients were CTC positive. An increase in CTC-positive patients was detected with increased tumor staging (Figure 3H). The mean number of CTCs was 4.85 and the median was 3.0. 

Patients with stage I or II disease harbored CTCs which were either enriched by positive selection (stage I and II) or showed CTCs in both positive and size dependent enrichment (stage II), hinting at a high expression of cell surface markers. Stage IV patients showed positivity in all enrichment approaches, which was also reflected in treatment naïve patients (Figure 3I). In accordance with the finding that RAS/RAF activating mutations result in a higher expression of melanoma surface markers (Figure 1), we detected more RAS/RAF mutated cells in the cellular subpopulation, which was enriched by positive selection, in comparison to the Parsortix™ enriched subpopulation. 

Patient 1 presented with an NRAS^Q61K^ primary melanoma and a positive sentinel lymph node biopsy at the time of the first liquid biopsy (Figure 3B–G). At the same time, a CSPG4/MCAM positive CTC was detected, containing a NRAS^Q61PL^ mutation. At week 4, the patient developed a lymph node metastasis and satellite metastasis, that were treated by surgical resection. After 62 weeks, we detected two marker positive CTCs, which contained a NRAS^Q61RL^ and a BRAF^V600E^ mutation and a surface marker negative CTC, without any RAS/RAF driver mutation. At week 74, the patient clinically relapsed (subcutaneous metastasis, SC). Between weeks 78–82, the SC metastasis was treated by radiotherapy, resulting in a complete response. At week 98, CTC analysis revealed one marker negative and RAF/RAS negative CTC. The patient relapsed at week 128, and was treated successfully with Pembrolizumab (PD-1 inhibitor). Note that CT scans, performed at weeks 12, 50, and 79, did not show any sign of metastasis or progression and LDH (lactate dehydrogenase) levels did not reflect the recurrence of the metastasis. S100 levels were elevated throughout the complete follow up period, thus limiting its predictive power, although this could possibly be helpful in the detection of minimal residual disease.

### 2.4. RNA Expression Pattern on Selected CTCs

Since we aimed to develop a CTC enrichment method which allows for the thorough analysis of melanoma CTC subpopulations, we tested the feasibility of the method for the analysis of RNA expression on single cell level. We first performed a pathway analysis in the TCGA cohort, comparing RAS/RAF mutated versus non-mutated melanomas. Using Cytoscape and ClueGO, a Go-term analysis of significantly upregulated genes (lgFC > 2, adj. *p*-value < 0.05) in the RAS/RAF cohort was performed. As expected, Go-terms, which are associated with the MAPK pathway, were significantly enriched (included in the “positive regulation of cell communication” cluster) (Supplementary Figure S1A). Interestingly, cell chemotaxis, which plays a major role in metastasis, was overrepresented as well. We next enriched CTCs from Patient 4 (BRAF^V600E^, stage IV) within 4 h of blood withdrawal, using the combined approach, but refraining from the use of fixation methods. In total, three marker positive and two marker negative cells were detected. An analysis of genes present in the Go-term “regulation of cell motility” by qRT-PCR, showed that marker positive cells indeed showed the enrichment of these genes (Supplementary Figure S1B).

### 2.5. CTCs and ctDNA Provide Synergistic Clinical Information

**Figure 4:**
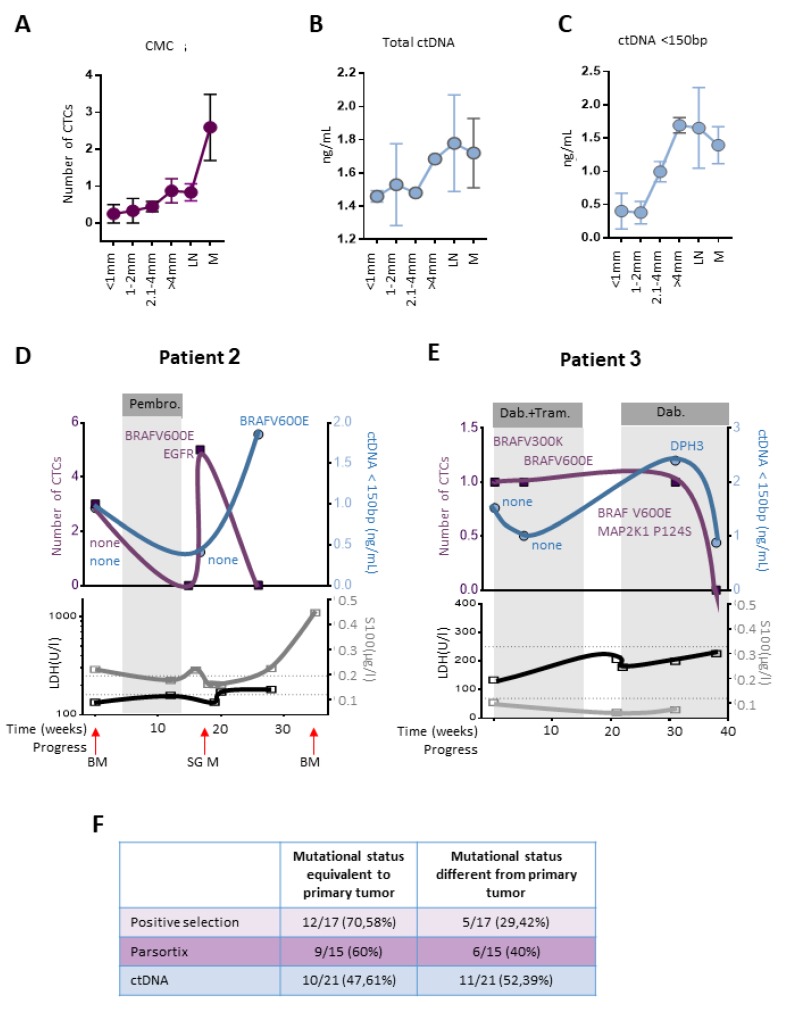
Comparison of CTC and ctDNA detection (**A**–**C**) CTCs, total ctDNA and ctDNA < 150 bp detected in patients with primary tumors (Breslow depth), lymph node metastasis (LN) and distant metastasis (M) (**D**,**E**) Time course of CTC detection, ctDNA, mutations detected in both CTCs and ctDNA, LDH and S100 serum levels in patients 2 and 3. (**F**) Mutational status for each detection method compared to the primary melanoma.

Information regarding how useful ctDNA might be for the stratification of melanoma patients, and whether ctDNA provides additional or congruent information in comparison to CTCs, is still sparse, and was therefore scrutinized. The detection of ctDNA against the normally occurring background of cell-free DNA is challenging. One possible solution might be the characterization of ctDNA fragment size. ctDNA has been reported to be overrepresented in the fraction below 150 bp [23]. Since it is conceivable that the amount of recovered ctDNA tumor depends on the tumor burden, we compared the number of CTCs, ctDNA > 150 bp and ctDNA < 150 bp in patients, with regards to the Breslow thickness of the primary tumor, or the existence of a lymph node or systemic metastasis (Figure 4A–C). 

CTC counts did not dramatically change between tumors below 1 mm and between 1–2 mm, however, an increase in the average number of detected cells was seen in tumors with a Breslow thickness above 2.1 mm, which was further increased in patients with systemic disease. Total ctDNA showed only a slight increase with increasing tumor thickness. ctDNA concentration below 150 bp allowed a comparable discrimination between primary tumors below and above 2 mm. Thus, both CTC count and ctDNA < 150 bp appear to be a promising tool to predict tumor burden in our cohort.

Patient 2 was a metastatic melanoma patient, without any detected driver mutations in the primary tumor (Figure 4D). At the time of first analysis, the patient presented a metastasis in the bone and the suprarenal gland (SG). At the same time, three CTCs were detected, and a ctDNA < 150 bp concentration of 0.9 ng/mL was measured. Both CTCs and ctDNA did not contain any driver mutations. The patient received Pembrolizumab, starting in week 4, and showed a partial response (bone). Meanwhile, the patient developed a pancreatic lesion. The PET-CT (positron emission tomography-computed tomography) confirmed a pancreatitis (possible side effect of Pembrolizumab), without any sign of new metastasis. At week 16, zero CTCs were detected. Two weeks later, we were able to detect five CTCs in this now-untreated metastatic melanoma patient, whereas the concentration of ctDNA < 150 bp was reduced, compared to the initial values. The PET-CT from the same day showed a progress of the SG metastasis and a new bone metastasis. Targeted sequencing of four out of the five CTCs revealed two BRAF^V600E^ and one EGFR^l491M^ mutation. At week 28, no CTCs were detected; however, ctDNA < 150 bp increased to ~2 ng/mL and confirmed the development of a BRAF^V600E^ mutated tumor. The patient progressed in week 36 (SG metastasis). Note that, in this case, LDH and S100 levels were poor markers for disease progression. S100, however, was dramatically increased at week 36.

Patient 3 was diagnosed with a BRAF^V600K^ positive melanoma in 2012. After a recurrence in 2013 and 2014, and LN metastasis and lung metastasis in 2015, treatment with Dabrafenib (BRAF inhibitor) and Trametinib (MEK inhibitor) resulted in a complete remission. One year later, in weeks 0, 5 and 31, one CTC was detected at each time point (Figure 4E). Note that, at week 11, the patient was diagnosed with schwannoma. Targeted sequencing showed BRAF mutations in all detected CTCs. A shift from BRAF^V600K^ to BRAF^V600E^ and later BRAF^V600K^ plus MAP2K1^P124S^ was found. At week 38, no CTCs were detected. In comparison, ctDNA < 150 bp was elevated at the time point of the initial blood draw, decreased at 5 weeks, and increased again at week 31. No mutations were found at either 0 or 5 weeks. At week 31, a DPH3 mutation was detected. The patient relapsed at week 118, with an upper arm metastasis. 

Melanomas have been known to quickly adapt their mutational pattern, in response to environmental and therapeutic pressure. Here, we tested whether mutations found in liquid biopsies of metastatic patients differed from the mutational status of the tissue derived from the primary tumor (reported by the department of pathology). The initial mutational status was recovered in 47.61–70.58% of all cases. Importantly, novel driver mutations were detected in 29.42–52.39% of all samples (Figure 4F).

### 2.6. CTCs, ctDNA < 150 bp and LDH Predict Clinical Outcome

**Figure 5:**
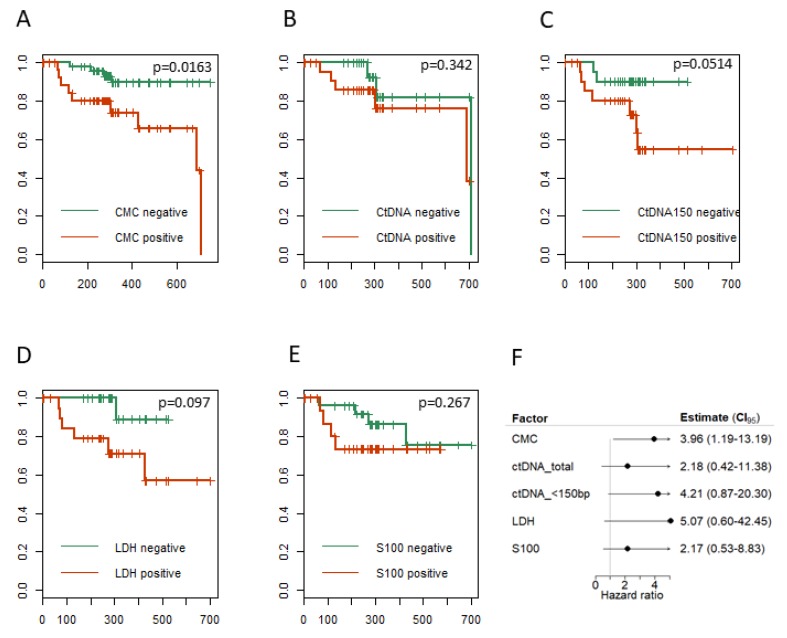
Prognostic value of liquid biopsy markers (**A**–**E**) Kaplan–Meier curves for each marker in the tested melanoma patient cohort. (**F**) Hazard ratio.

We next analyzed whether stratification of patients by the existence of CTCs or a ctDNA concentration higher than 2 ng/mL for ctDNA > 150 bp, or 0.5 ng/mL for ctDNA < 150 bp, would predict patient survival. The cut-off values were chosen based on values detected in blood from healthy donors. Kaplan–Meier curves demonstrate that patients with detectable CTC (≥1 CTC), ctDNA < 150 bp and LDH positive patients show a worse outcome than marker negative patients (Figure 5A–E). In a cox-proportional hazards regression analysis, adjusted for stage, age, gender and treatment, the hazard ratio for LDH was calculated to be 5.07, followed by ctDNA < 150 bp (4.21) and CTCs (3.96). ctDNA > 150 bp and S100 were not found to significantly alter the HR in melanoma patients (Figure 5F). Note that Patients 2 and 3 (Figure 4D–E) were selected based on interesting and representative clinical courses, and are not representative of the HR values calculated in Figure 5F. 

## 3. Discussion

Our work showed that surface marker expression on melanoma cells is dependent on their mutational status. Furthermore, we demonstrated that a combined analysis of ctDNA and CTCs predicted relapse earlier than imaging, and was more accurate than serum LDH or S100 in a subset of patients. Interestingly, we were able to detect “private” mutations on CTCs and ctDNA, that were not revealed in the random bulk analysis of the primary tumor. 

In the present study, we have analyzed melanoma-associated cell surface markers in relation to the mutational status of the melanoma cells. We found a larger proportion of surface marker-positive cells (e.g., CSPG4/MCAM) in the RAS/RAF-mutated cohort compared to the non-RAF/RAS mutated cohort. Thus, we conclude that the commonly employed enrichment of CTCs based on surface marker expression might be biased and could lead to the loss of subsets of tumor cells lacking the appropriate mutational status. Consequently, we developed our own CTC approach, combining a marker dependent and marker independent detection method. We combined positive selection, using CSPG4 and CD146 MACS microbeads and Parsortix™, in order to prevent the loss of marker-negative tumor cells. However, one limitation of our study is the focus on RAS/RAF mutations. Even though RAS/RAF mutated tumors present the majority of mutated melanomas, further research will have to be conducted to test whether alternative driver mutations might also be represented by specific marker expression. 

Overall, 32% of patients were CTC-positive. An increase in CTC-positive patients was detected with increased tumor staging. Enrichment of melanoma CTCs was challenging, due to intra-patient heterogeneity and inter-patient heterogeneity, including different disease stages, subtypes and therapy regimes, as reflected in our patient characteristics (Figure 3A). 

It was previously suggested that ctDNA is more accurate in predicting response to targeted therapy and immunotherapy than serum LDH [24,25]. An increased quantity of ctDNA can be found in the circulation of cancer patients [26]. ctDNA is released from tumor cells via different mechanisms, such as apoptosis, necrosis and secretion [15,26]. The most common mutation in melanoma BRAF can be detected in the ctDNA of melanoma patients and has been shown to be useful in monitoring patients [27]. Sensitive technical strategies for ctDNA detection include ddPCR and BEAMing [6,15,25,28]. Here, we have used a rapid and cost-effective approach for ctDNA analysis, which is based on mass spectrometry, can be used for sensitive multiplex analyses, and requires no bioinformatics. To our knowledge, there is only one previous report using this approach for ctDNA detection in melanoma patients [29]. The panel achieved 92% concordance with ddPCR for the detection of BRAFV600E in ctDNA and was capable of measuring increased levels of mutation in metastatic melanoma patients undergoing therapy prior to radiological progression [29].

Finally, we established a dual approach to detect ctDNA and CTCs and showed proof-of-principle data on two index patients. Patient 2 partially responded to anti-PD1 treatment with Pembrolizumab for 8 weeks and developed pancreatitis; treatment was then discontinued, and the patient relapsed. Intriguingly, CTCs obtained at the time of relapse reveal both a BRAFV600E and EGFRI491M mutation, suggesting a potential benefit from targeted therapy. ctDNA < 150 bp was not detected at this time point, but later, in association with more severe disease progression. Thus, the combined assessment of CTCs and ctDNA can provide complementary information. Patient 3 revealed both CTCs and ctDNA < 150 bp, even during a period of complete clinical remission, in response to BRAF/MEK inhibition. A BRAF and MAPK activating mutation positive CTC was detected during the time of BRAF inhibitor treatment, possibly the first indication of an emerging resistance. 

Serum proteins have been frequently used as biomarkers in melanoma in the past. LDH is the only blood-based biomarker implemented in the AJCC melanoma staging system, since the elevated serum LDH is associated with significantly decreased survival in patients with stage IV disease [30]. Nonetheless, LDH is not specific to melanoma or other malignancies; LDH activity can, for example, increase in response to tissue injury of the liver or heart [31]. In addition, levels of serum S100B can indicate clinical response to treatment [32,33]. However, S100 proteins also show an elevated expression in cardiovascular, neurological and inflammatory diseases [33]. Thus, the interpretation of therapy responses using LDH and S100 can be limited, which is reflected in our data. When Patient 2 relapsed at week 18, five CTCs were detected. Neither ctDNA, LDH or S100 levels were elevated. For Patient 3, ctNDA < 150 bp was elevated at week 31, when a DPH3 mutation was detected, possibly a first indication of relapse, which occurred at week 118. At that time point, LDH or S100 levels were within reference values. The relevance of DPH3 mutations in the process of carcinogenesis remains to be determined [34,35]. However, it is noteworthy that DPH3 over-expression was shown to promote cellular invasion and metastasis in murine melanoma cells in vivo, whereas silencing of DPH3 reduced development of metastasis [36]. 

CTC count and ctDNA < 150 bp appear to be promising tools to predict tumor burden in our cohort. Kaplan–Meier curves demonstrated that patients with detectable CTCs, ctDNA < 150 bp and LDH positive patients show a worse outcome than composite marker-negative patients. This finding is in line with previous studies, where ctDNA levels provide an accurate prediction of tumor response and overall survival in patients treated with PD1 inhibitors [37]. Additionally, baseline ctDNA levels have been found by another group to be significantly associated with progression-free survival in patients treated with BRAF inhibitor therapy [15], and CMCs have shown prognostic value concerning survival in previous studies [38,39,40].

According to the European Society for Medical Oncology (ESMO) guidelines for melanoma, mutation testing on biopsies for treatable mutations is mandatory in patients with advanced disease, to select the appropriate systemic therapy. In cases of inaccessible metastases, liquid biopsy might become a potential approach to guide therapy decisions. The initial mutational status (i.e., mutations in BRAF, NRAS) of the primary tumor was recovered in CTC and ctDNA in 47.6–70.6% of all cases. Importantly, private mutations, not detectable on the primary tumor of the same patient mutations, were detected on CTCs and ctDNA in 29.4–52.4% of all samples, suggesting that liquid biopsy can provide complementary information to analysis of tissue biopsies. Previous studies focusing on BRAF mutations found a concordance between plasma ctDNA and tumor BRAF mutations of 75–76% [41,42]. The detection of mutations, which are not present in the primary tumor, might help to assess tumor heterogeneity and track clonal tumor evolution in individual patients. 

## 4. Material and Methods

### 4.1. Patient Samples 

A total of 100 patients were recruited from January 2014 until November 2016 at the Clinic for Dermatology, University Hospital Hamburg-Eppendorf and the Clinic for Dermatology, Elbe-Klinikum-Buxtehude. A total of 84 patients with malignant melanoma fulfilled the inclusion criteria (written informed consent, blood draw, stage I–IV). Patients were staged according to the TNM classification for malignant melanoma (AJCC 2009). Patients of all stages, aged 21–88 years, with cutaneous, uveal, acral and melanoma of unknown primary were included. Blood samples obtained from healthy donors served as a negative control. Blood was drawn into ethylene diamine tetra-acetic acid (EDTA) tubes. The number of CTCs was determined per EDTA-tube (approx. 7.5 mL) of peripheral blood. Written informed consent was obtained from all participants prior to the blood draw, in accordance with the principles and patient rights laid down in the declaration of Helsinki. All laboratory procedures have been approved by the Ethics Committee Hamburg (ethics application PV3779). Our study adheres to the REMARK criteria [43]. Lactate dehydrogenase (LDH) and S100B levels were measured independently by the Department of Pathology, University Hospital Hamburg-Eppendorf. 

### 4.2. Tumor Cell Enrichment

To identify the most suitable method to detect different CTC subpopulations, 25 BRAF^V600E^ positive SKMEL28 cells and 25 NRAS/BRAF wildtype MeWo cells (kindly provided by Prof. Dr. med. Udo Schumacher, UKE, Germany) were both spiked into 7.5 mL of blood from healthy donors. Both cell lines were purchased via ATCC, and continuously monitored every three months by STR Profiling and mycoplasma testing (PCR). Subsequently, marker dependent (Cellsearch^®^, MACS) and independent (Leucosep^TM^, Parsortix^TM^, MACS) [44,45] enrichment methods were tested. 

#### 4.2.1. Marker-Dependent Approach

1. MACS(+)

Peripheral blood samples were collected in EDTA tubes (Sarstedt, Nürmbrecht, Germany). Plasma separation was performed as described below (ctDNA analysis). Density gradient centrifugation using Leucosep™ tubes (Greiner Bio-One, Kremsmünster, Österreich) and Ficoll-Paque™ media (GE Healthcare, Chicago, IL, USA) was performed to isolate the peripheral blood mononuclear cells (PBMCs) (800× *g*, 10 min). The mononuclear cell fraction was transferred to a new 50 mL tube, washed once and centrifuged for 15 min at 300× *g* in order to form a cell pallet. Cells were resuspended in 200 µL DPBS (Gibco^®^ by Life Technologies™, Carlsbad, CA, USA) and either stored at −80 °C with 100µl DMSO (Serva, Heidelberg, Germany) and 200 µL FCS or immediately processed.

The cells were fixed by adding 700 µL 0.5% paraformaldehyde solution and centrifuged for 10 min at 300× *g*. After resuspension in 300 µL MACS Buffer (Miltenyi Biotec, Bergisch Gladbach, Germany), the tumor cells were magnetically labelled by adding 20 µL anti-CD146 MicroBead Kit (CD146 MicroBeads and FcR Blocking Reagent, Miltenyi Biotec, Bergisch Gladbach, Germany) and 20 µL Anti-Melanoma (CSPG4) MicroBeads (Miltenyi Biotec, Bergisch Gladbach, Germany) to the cell pellet and incubated at 4 °C for 30 min. After centrifugation (12 min 300× *g*), 1 mL MACS Buffer was added and the cell suspension was inserted into a MACS separation column (Miltenyi Biotec, Bergisch Gladbach, Germany) that had been equilibrated with MACS buffer. Magnetically labelled cells adhered to the column, while the unlabeled cells passed through. The MACS column was removed from the magnetic field. The labelled tumor cells were flushed from the column with 3 mL MACS Buffer, and additional 3 mL by force. Finally, the cell suspension was centrifuged for 4 min at 1200× *g* in order to secure cells on a glass slide.

2. Cellsearch^®^

Peripheral blood was collected in one CellSave™ tube (approx. 10 mL) (Menarini Silicon Biosystems, Bologna, Italy). Following the manufacturer’s protocol, the CellSearch^®^ CMC assay (Menarini Silicon Biosystems, Bologna, Italy) was performed within 24 h after sample collection. Circulating melanoma cells were defined as CD146^+^, CSPG4^+^, CD45^−^, CD34^−^, nucleated (DAPI^+^) cells, as previously described [38].

#### 4.2.2. Marker-Independent Approach

1. Leucosep^®^

Peripheral blood samples were collected in EDTA tubes. After performing plasma separation of the whole blood sample (described below) density gradient centrifugation with Leucosep™ tubes and Ficoll-Paque™ media was used to isolate the peripheral blood mononuclear cells (PBMCs) (800× *g*, 10 min). The mononuclear cell fraction was transferred to a new 50 mL tube, washed once and centrifuged for 15 min at 300× *g*, in order to form a cell pellet. After resuspending the cells in PBS, they were transferred to glass slides by cytospin centrifugation. 

2. Negative selection/MACS(−)

Mononuclear cells were prepared as described above (positive selection). CD45 positive cells were depleted from the sample, using anti-CD45 magnetic beads, according to the manufacturer’s protocol (Miltenyi, Bergisch Gladbach, Germany). 

3. Marker-independent CTC enrichment (Parsortix^TM^ device)

Parsortix™ is a size and deformability-based method that allows for marker-independent CTC enrichment (Angle Plc, Guilford, UK). Cells were separated according to their size and deformability (final separation gap 8 µm), using a disposable cassette, according to our previous work [45]. 

#### 4.2.3. Combined Approach (MACS and Partsortix^®^)

After adhesion of the magnetically labelled melanoma cells to the column, the column (LS) was washed with 3 mL MACS Buffer (Miltenyi, Bergisch Gladbach, Germany) and the flow-through (4 mL, marker negative melanoma cells and other mononuclear cells) was collected in a Parsortix™ tube and subsequently enriched by the Parsortix™ method. For the isolation of marker positive cells, the MACS column (Miltenyi, Bergisch Gladbach, Germany) was removed from the magnetic field. The labelled tumor cells were flushed from the column with a 6 mL MACS Buffer.

### 4.3. Immunofluorescence Staining 

After enrichment, cells were transferred to the cytospin (max. 1 million per slide, 3 min 1200 r.p.m.) and dried overnight. After fixation with 0.5% paraformaldehyde solution for 10 min, cells were stained for surface markers CSPG4 (anti hNG2/MCSP, R&D Systems, Minneapolis, MN, USA) and MCAM (anti-CD146 monoclonal antibody, Merck KGaA Darmstadt, Germany) (positive markers) or cytoplasmic S100 (Anti-S100, Dako Denmark A/S, Glostrup, Denmark) (to enable detection of surface marker negative cells), the common leukocyte antigen CD45 (FITC anti-human CD45, BioLegend^R^, San Diego, CA, USA) (negative marker), and the nuclear dye DAPI. The slide was incubated with the respective antibodies for 1 h at room temperature, or overnight at 4 °C. Enriched cells were quantified by fluorescence microscopy. Morphologically intact NG2+/MCAM+/CD45−/DAPI+ cells were defined as CTCs and picked with a micromanipulator. Single cells were stored at −80 °C for future amplification and mutational analysis.

### 4.4. Whole Genome Amplification

Whole genome amplification (WGA) on isolated CTCs was performed using the Ampli1 Kit (Silicon Biosystems, Castel Maggiore, Italy), according to the manufacturer’s instructions. Quality of the WGA product was analyzed using the Ampli1 QC Kit (Silicon Biosystems, Castel Maggiore, Italy).

### 4.5. cfDNA Extraction

Blood samples were collected in EDTA tubes, stored at RT and processed within 6 h. Shipped blood samples were stored in StreckTubes and processed within 36 h. In order to isolate the plasma from the whole blood, the samples were double centrifuged for 10 min at 300× *g*. Plasma was transferred to a new tube and centrifuged at 2000× *g* for 15 min to remove cellular debris. Plasma aliquots were stored at −20 °C/−80 °C. Cell-free DNA (cfDNA) was isolated from 1–5 mL plasma with the QIAamp Circulating Nucleic Acid Kit (Qiagen, Hilden, Germany), according to manufacturer’s instructions, with a final elution volume of 40 µL. 

### 4.6. Quantification and Size Fragment Distribution of cfDNA

The concentration of cfDNA was determined using a NanoDrop Spectrometer ND-1000 (Thermo Fisher Scientific, Waltham, MA, USA) with a sample volume of 1 µL. Fragment distribution was assessed using the 4200 TapeStation device, using the High Sensitivity D5000 ScreenTape Assay with 1 µL sample, and 5 µL High Sensitivity D5000 Sample Buffer (Agilent, Santa Clara, CA, USA). 

### 4.7. Mutational Analysis

Mutational analysis was performed using the UltraSEEK™ Melanoma Panel v1.0 (Agena Bioscience, Hamburg, Germany), interrogating 61 clinically relevant variants across 13 genes, including BRAF, NRAS, KIT and MAP2K1, detected at as low as 0.1% minor allele frequency. Reactions were performed as described before [46]. In brief, PCR (45 cycles) was followed by shrimp alkaline phosphatase treatment and single base primer extension, using biotinylated ddNTPs specific for the mutant alleles. After capture of the extended primers using streptavidin-coated magnetic beads, a cation-exchange resin was added for cleaning, and 10–15 nL of the reaction was transferred to a SpectroCHIP^®^ Array (a silicon chip with pre-spotted matrix crystals) using an RS1000 Nanodispenser (Agena Bioscience). Data were acquired via matrix-assisted laser desorption/ionization time-of-flight mass spectrometry, using a MassARRAY Analyzer 4 (Agena Bioscience, Hamburg, Germany). After data processing, a spectrum was produced with relative intensity on the y-axis and mass/charge on the x-axis. Typer Analyzer software was used for data analysis and automated report generation. Sanger sequencing was performed to verify mutations detected by the UltraSEEK™ Melanoma Panel, and only mutations which were detected in both assays (98%) were used for further analysis. 

### 4.8. RNA Analysis 

CTCs were isolated within 4 h of blood withdrawal. cDNA synthesis and amplification were performed using the SuperScript II Kit (Thermo Fisher Scientific, Waltham, MA, USA), according to the manufacturer’s recommendations. 

### 4.9. Bioinformatical and Statistical Analysis

TCGA data was last accessed via the following webpage in November 2017—http://firebrowse.org/. Differential analysis was performed using the R packages edgR and Limma. Go-term analysis of genes upregulated in the RAS/RAF group FC > 2 adj. *p*-value < 0.05 was performed using the ClueGo app in Cytoscape. Single cell analysis of the Tirosh et al. dataset (GSE72056) was performed using the R packages SingleCellExperiment. Data were plotted using ggplot2.

The distribution of disease specific survival was estimated using the method of Kaplan–Meier. Median values for the cox regression model for the distributions of the HRs and P values are reported with 95% empirical. Analysis was performed using the R-packages survminer.

Statistical analysis was performed using GraphPad Prism Software (GraphPad Software Inc., La Jolla, CA, USA). All datasets are represented as mean ± SEM and were analysed either by ANOVA and Tukey’s or Holm–Sidak’s multiple comparison correction. Statistical significance was considered at *p*-values of *p* < 0.05.

## 5. Conclusions

In summary, analysis of CTCs in combination with ctDNA provides complementary information, beyond the current serum biomarkers LDH and S100, which might help to personalize targeted and immunotherapies for melanoma patients in the future. However, the present findings need to be validated in larger future studies before implementation into clinical practice.

## Supplementary Materials

The following are available online at https://www.mdpi.com/2072-6694/11/11/1685/s1, Figure S1: Pathway analysis in the TCGA cohort, Table S1: Overview CTC positive patients.
