# Supplementary material for: Intra-Patient Heterogeneity of Circulating Tumor Cells and Circulating Tumor DNA in Blood of Melanoma Patients
Source: Cancers (Basel). 2019 Oct 29;11(11):1685. doi: 10.3390/cancers11111685 (PMC6896052; doi:10.3390/cancers11111685)
Supplement: Supplementary file 1 [file cancers-11-01685-s001.pdf]

Supplementary Materials: Intra-patient heterogeneity of circulating tumor cells and circulating tumor DNA in blood of melanoma patients

Katharina Gorges, Lisa Wiltfang, Tobias M. Gorges, Alexander Sartori, Lina Hildebrandt, Laura Keller, Beate Volkmer, Sven Peine, Anna Babayan, Ingrid Moll, Stefan W. Schneider, Sören Twarock, Peter Mohr, Jens W. Fischer and Klaus Pantel

Table S1. Overview CTC positive patients.

Patient_ID	Sex	Age	Melanoma_Type	Mutational_Status	TNM	Survival_Status	Detected_CTCs	Ulceration	S100 (µg/L)	LDH (U/L)	Number_of_ Follow_Ups
patient_1	m	50	SSM	unknown	T1bN0M0	alive	2	No	0.021	227	1
patient_2	m	46	nodular	BRAF	T3bN2cM0	alive	3	Yes	0.034	NS	1
patient_3	m	58	NS	unknown	T3aN0M0	alive	2	No	0.039	272	1
patient_4	m	57	unknown	BRAF	TxN0M1c	alive	4	NS	0.058	143	5
patient_5	m	65	desmoplastic	BRAF	NS	alive	1	NS	0.061	NS	2
patient_6	f	71	unknown	none	TxN0M1c	alive	8	NS	0.089	290	4
patient_7	m	39	unknown	unknown	TxNxM1c	dead	4	NS	0.09	804	1
patient_8	f	88	cutaneous	BRAF	T2aN1aM1b	alive	1	No	0.11	211	1
patient_9	m	74	cutaneous	BRAF	T4N2bM1a	alive	3	NS	0.122	269	4
patient_10	f	61	nodular	BRAF	T3aN2cM0	alive	1	No	0.151	NS	1
patient_11	m	88	cutaneous	unknown	T2aN2cM0	dead	2	No	0.254	NS	1
patient_12	m	69	unknown	NRAS	TxNxM1a	alive	3	NS	0.27	217	1
patient_13	f	69	cutaneous	BRAF	T3bN2cM1c	alive	1	Yes	0.394	NS	1
patient_14	m	46	nodular	BRAF	T4aN3M1c	dead	2	No	0.769	213	1
patient_15	f	NS	cutaneous	BRAF	T4aN2cM0	alive	1	No	0.9	216	1
patient_16	m	66	cutaneous	none	T4bN0M1c	dead	1	Yes	1.52	219	1
patient_17	m	74	unknown	BRAF	TxNxM1c	dead	3	NS	9.463	680	1
patient_18	f	52	cutaneous	BRAF	T3bN1aM1c	dead	62	Yes	19.71	493	1
patient_19	f	34	SSM	NRAS	T1bN1aMx	alive	3	Yes	NS	160	1
patient_20	m	44	uveal	unknown	TxN0M1c	alive	5	NS	NS	166	2
patient_21	m	76	SSM	unknown	T1aN0Mx	alive	1	No	NS	NS	1
patient_22	m	76	unknown	BRAF	TxNxM1a	alive	3	NS	NS	NS	2
patient_23	m	62	cutaneous	BRAF	T1bN2M1c	dead	1	Yes	NS	NS	1
patient_24	m	58	nodular	BRAF	T4bN3M1a	alive	1	NS	NS	NS	4
patient_25	f	76	amelanotic	unknown	T4bN0M0	alive	4	NS	NS	NS	4
patient_26	f	85	amelanotic	NRAS	T4bN3M0	dead	3	NS	NS	NS	4

patient_27	f	81	nodular	NRAS	T4bN0M0	dead	6	NS	NS	NS	3

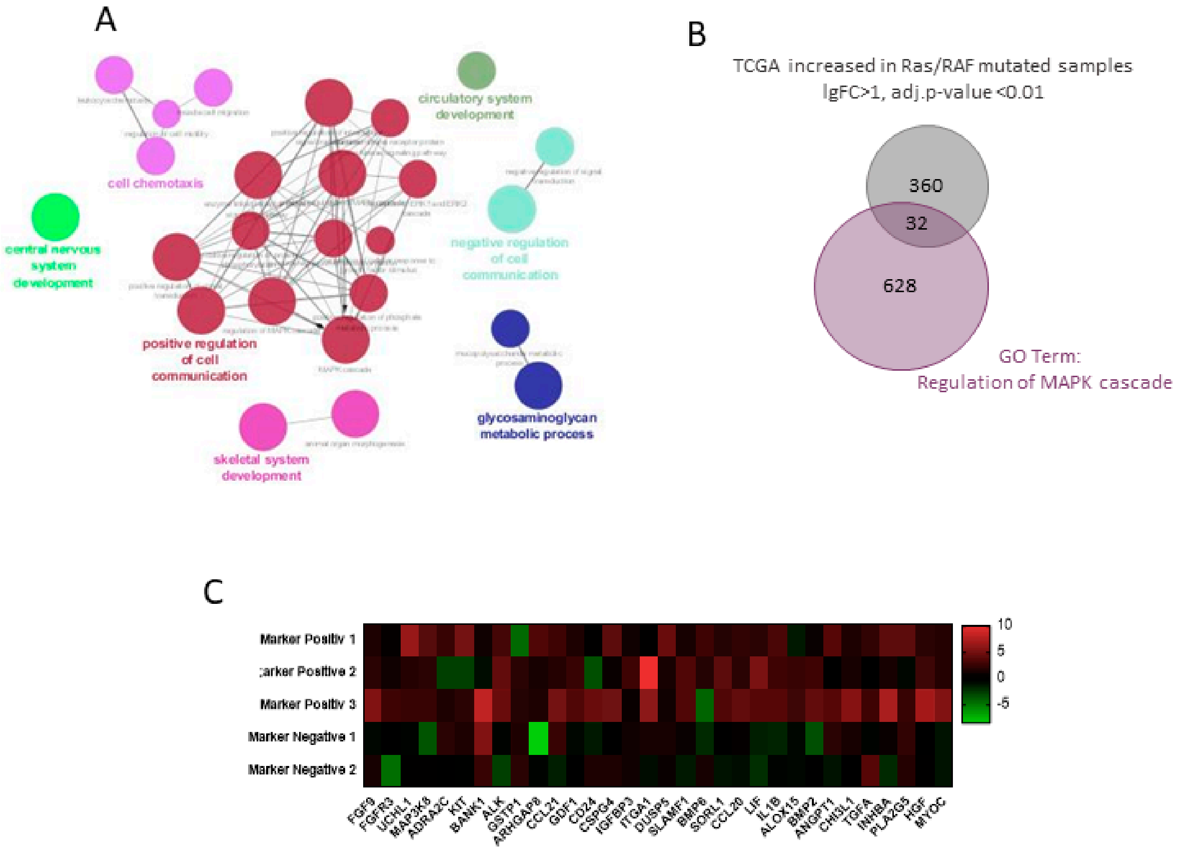

Figure S1. Pathway analysis in the TCGA cohort. mRNA analysis of single cells (**A**) GO-Terms enriched in Ras/RAF mutated samples from the TCGA cohort. Analysis was performed using Cytoscape. (**B**) Venn-diagram of genes significantly increased in Ras/RAF mutated samples compared to non-mutated samples (TCGA) and genes listed in the GO-term "Regulation of MAPK cascade". (**C**) mRNA expression analysis of marker negative and marker positive single cells.
